# Dual Targeting and Retrograde Translocation: Regulators of Plant Nuclear Gene Expression Can Be Sequestered by Plastids

**DOI:** 10.3390/ijms130911085

**Published:** 2012-09-06

**Authors:** Kirsten Krause, Svenja Oetke, Karin Krupinska

**Affiliations:** 1 Department of Arctic and Marine Biology, University of Tromsø, Tromsø 9037, Norway; E-Mail: kirsten.krause@uit.no; 2 Institute of Botany, University of Kiel, Olshausenstrasse 40, Kiel 24098, Germany; E-Mail: soetke@bot.uni-kiel.de

**Keywords:** chloroplasts, protein targeting, retrograde signals, WHIRLY1

## Abstract

Changes in the developmental or metabolic state of plastids can trigger profound changes in the transcript profiles of nuclear genes. Many nuclear transcription factors were shown to be controlled by signals generated in the organelles. In addition to the many different compounds for which an involvement in retrograde signaling is discussed, accumulating evidence suggests a role for proteins in plastid-to-nucleus communication. These proteins might be sequestered in the plastids before they act as transcriptional regulators in the nucleus. Indeed, several proteins exhibiting a dual localization in the plastids and the nucleus are promising candidates for such a direct signal transduction involving regulatory protein storage in the plastids. Among such proteins, the nuclear transcription factor WHIRLY1 stands out as being the only protein for which an export from plastids and translocation to the nucleus has been experimentally demonstrated. Other proteins, however, strongly support the notion that this pathway might be more common than currently believed.

## 1. Introduction: Plastids as Sensors and Efficient Communicators of Environmental Conditions

Plastids are the characteristic organelles of plant cells. They are best known for their photosynthetic function and as factories producing numerous compounds for metabolism. In higher plants chloroplasts develop from undifferentiated proplastids in meristematic cells or from etioplasts, which might differentiate from proplastids when plants germinate in darkness. The characteristic structural and functional plasticity of the plastids depends on the tissue, the developmental age and the environmental situation the plant is experiencing. In other words, the differentiation state of plastids is the result of diverse endogenous and exogenous influences/factors. Due to their sessile way of life, plants need efficient receptors for diverse abiotic and biotic stimuli to respond continuously to changes in the environment.

Chloroplasts play a central role in sensing the environmental situation and executing adaptive responses of plants [1]. The functionality of their photosynthetic apparatus depends on various factors including nutrient supply, light and temperature. Subtle changes in light quality and intensity can have tremendous effects on the redox state of the photosynthetic apparatus [2,3] and can also lead to production of specific reactive oxygen species such as singlet oxygen and superoxide anions [4]. Such retrograde signals are part of the chloroplast-to-nucleus communication (retrograde signaling) and are known to induce specific changes in nuclear gene expression [5]. The *de novo* generation of some of these signals in the chloroplast might need considerable time to achieve certain threshold levels necessary for induction of signaling. This has recently been corroborated by measurements for several different signaling compounds upon high light stress (3′-phosphoadenosine 5′-phosphate [6], methylerythrol cyclodiphosphate [7], β-cyclocitral [8]). It is, therefore, unlikely that they are involved in immediate responses to sudden stressors such as wounding and attacks by pathogens, which plants might be exposed to. Nevertheless, it became obvious in recent years that plastids are involved in recognition of pathogens [9] and in responses to wounding [10,11]. The mechanisms range from recognition of the pathogen to the synthesis of signaling compounds typically involved in the plants’ responses to biotic (and abiotic) stressors: salicylic acid (SA), jasmonic acid (JA) and abscisic acid (ABA) [1].

In this review the role of proteins in chloroplast-to-nucleus communication is discussed. Increasing evidence indicates that chloroplasts indeed possess numerous proteins, which have also been detected in the nucleus and/or the cytoplasm. Generally, one needs to distinguish between proteins that are sequestered on the cytoplasmic face of the chloroplast envelope membrane and are released upon certain triggers by specific endopeptidases and proteins that are sequestered in the stroma of chloroplasts. Examples for the first group have been discussed before [12]. Recently, another such protein, the PHD transcription factor PTM was shown to accumulate in the nucleus after release from the plastid surface. There it activates the transcription factor ABI4, thereby providing a way to communicate the plastid status to the nucleus [13]. In contrast, the second group of intraplastidially stored proteins has potential access to plastid and nuclear DNA and is thus the only group of proteins that can directly be involved in the coordination of gene expression in both compartments. In the following chapters we will focus on this second group of dually targeted proteins.

## 2. The Concept of Compartment-Specific Protein Targeting Requires Revision

The original dogma that each polypeptide chain fulfils only one function has been replaced over the last two decades by the notion that many—if not most—proteins are bi- or even multifunctional. One of the reasons why some isoforms of many enzymes have not been discovered until fairly recently is that the secondary functions are exerted in other compartments than the initially attributed primary function [14]. A phenomenon of many such dual targeted proteins, which has further aggravated their analysis, is their uneven or “eclipsed” distribution between their different target compartments [15]. To achieve dual or multiple targeting of a protein product from a single gene, different strategies might be used. These include ambiguous signals that can be recognized by the import machineries of more than one compartment (e.g., mitochondria and plastids) and various forms of twin targeting where two or more distinct localization signals are encoded by the gene. Among the regulatory mechanisms that ensure the correct spatial and temporal distribution of the latter type of proteins, are alternative transcriptional or translational start sites, alternative splicing and post-translational modifications of subsets of the protein pool as reviewed by Krause and Krupinska [12].

Besides the dual targeting of proteins, which are synthesized *de novo*, other proteins might get translocated from their primary compartment to a secondary compartment. Such a release of proteins is well known for mitochondria at the onset of programmed cell death [16]. By comparison, speculations on protein export from chloroplasts have only been substantiated by first experimental evidence very recently [17].

## 3. A Growing Number of Genes Encode Proteins Targeted to More Than One DNA Containing Compartment

In 1998 Small and coworkers postulated that the multitude of shared activities connected to the expression and maintenance of the genetic information located in the nucleus and the organelles should entail the occurrence of a larger set of proteins shared by two or all three DNA containing compartments [18]. Although this hypothesis was feasible, at that time only one example, the carrot dihydrofolate reductase-thymidylate synthase (DHFR) [19], was published. Later on, many more examples were found. One extreme example for the postulated dual-targeting is the family of aminoacyl-tRNA-transferases where in *Arabidopsis thaliana* at least 15 members are shared between plastids and mitochondria [20].

Many dual targeting events initially escaped the attention of researchers because the isoforms of the corresponding proteins were described under different names and often in different species or were mistaken as paralogs. Some of the earliest known examples are the plastid RNA binding proteins cp29 and cp31 [21] and the MAR-binding filament like protein 1 (MFP1) [22] (Table 1). In plastids, cp29 as well as cp31 bind to RNAs [21] whereas in the nucleus they bind to DNA. There, cp29 functions as a transcriptional repressor of the pathogenesis related gene *PR-10a* under the name SEBF [23] while the nuclear isoform of cp31 (also termed STEP1) binds to telomeres [24]. In the case of MFP1, its appearance in speckles at the nuclear periphery was first interpreted as an indication for an exclusive localization in the nuclear envelope [25] and only later these speckles were correctly assigned to proplastids lining up at the periphery of the nucleus [26].

In most studies on dual or multiple targeting the molecular masses of the proteins in different compartments are unknown [12]. The same gene can give rise to the production of two proteins of different molecular masses when the gene has two transcription initiation sites as it has been shown for the DHFR dually targeted to plastids and the nucleus [19] (Table 1). In other cases, however, no evidence for alternative transcription start sites or splice variants was found. Two proteins might be synthesized from two alternative translation initiation sites of the same transcript or result from different processing of one precursor protein. The Win4 protein is an example for alternative translation initiation from one transcript. A 26 kD form localizes to the cytoplasm and nucleus whereas a 24 kD precursor protein is imported into plastids where it is processed to a 17 kD mature protein [40]. The two SWIB-4 proteins detected in chloroplast nucleoids and the nucleus, respectively, clearly derive from the same precursor. The nuclear form has the molecular mass of the precursor protein whereas the plastidic form has a lower molecular mass due to processing in the plastid [48].

In contrast, other proteins such as WHIRLY1 have the same molecular mass in two different compartments. Upon import into the plastids, WHIRLY1 is processed by cleavage of an *N*-terminal target peptide [34], resulting in a truncated mature protein. Recently, it was shown that the mature form is released from chloroplasts and accumulates in the nucleus [17], thus providing a novel form of dual targeting that involves retrograde translocation from the primary target compartment. This retrograde translocation explains why the nuclear isoform is of the same size as the mature chloroplast protein [33]. In the nucleus, WHIRLY1 fulfils various functions, among them the maintenance of telomere homeostasis [36] as well as the activation or repression of transcription reported for several genes that are involved in pathogen defense reactions [35,49].

Several proteins were suggested to be located in plastids and the nucleus, respectively, on the basis of bioinformatic predictions [43] and indirect experimental approaches such as the localization of GFP fusion proteins [47]. An interesting example for dual targeting are the SIB-1 and SIB-2 proteins known to interact with the SIGMA1 factor of plastid encoded RNA polymerase. Unexpectedly, both proteins were shown to interact with the WRKY33 transcription factor involved in pathogen response reactions [46]. Resistance to the necrotrophic pathogen *Botrytis cinerea* was compromised in *sib1* and *sib2* mutants, whereas resistance was enhanced in SIB1 over-expressing plants [46]. The authors concluded that for interaction with WRKY33, SIB-1 and SIB-2 might be translocated to the nucleus. However, another scenario is equally possible. WRKY33 was found among the subset of transcription factors having a prediction to be targeted to both plastids and the nucleus [43]. Although experimental evidence is still lacking, it might be possible that not SIB-1 and SIB-2 are located to the nucleus, but rather WRKY33 is located in plastids.

## 4. What is the Reason for Sequestration of Nuclear Proteins in Plastids?

**Figure 1:**
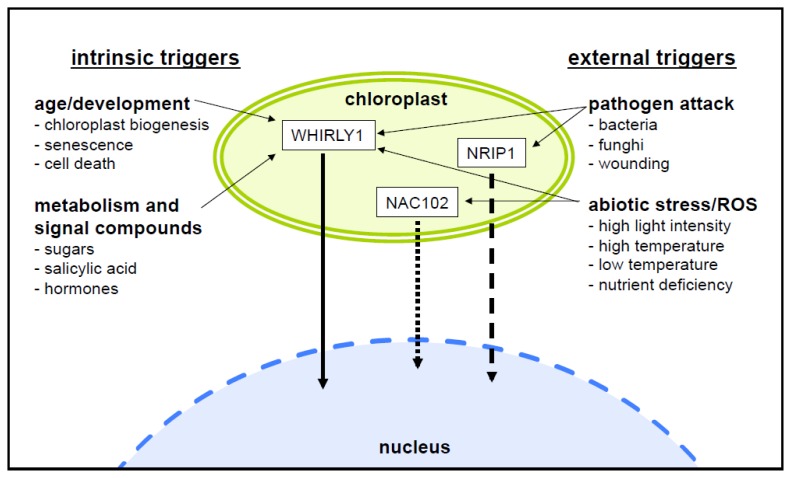
Chloroplasts are involved in the perception of intrinsic triggers controlling plant development and external cues and stresses from the abiotic and the biotic environment. A novel signaling pathway involving chloroplast located proteins that might be translocated to the nucleus in response to the diverse stimuli perceived by chloroplasts (see text) is depicted here. Whirly1 is the only protein for which a *bona fide* export has been shown to date (arrow with continuous line). This protein is involved in pathogen responses [35,52], but might also play a role in other situations. The NRIP1 protein appears to be released from plastids in response to infection to tobacco with the tobacco mosaic virus [45] but direct evidence for an export is yet missing (arrow with large dotted line). The transcription factor NAC102 is an intriguing candidate for the perception of oxidative stress in chloroplasts [47], but the possibility of its release remains to be investigated (arrow with small dotted line).

A subset of the dually targeted proteins described above and listed in Table 1 is involved in the reaction to biotic and abiotic factors, including pathogen defense, fitting with the notion that the plastids play a role as sensory organelles for environmental changes [1]. It is, therefore, logical to speculate that such proteins play a vital role in plastid-to-nucleus retrograde signaling. In contrast to other components involved in retrograde signaling (such as intermediates of plastid metabolic pathways, for example) proteins involved in gene regulation could be direct mediators of gene expression changes. The storage and release of proteins from plastids and their subsequent translocation to the nucleus could allow a fast response to changes in plastid-localized processes upon certain triggers (Figure 1). WHIRLY1 was first described as a transcriptional activator of the *PR10a* gene of potato in the nucleus [35]. Its binding to promoters of target genes was shown to most likely depend on a posttranslational activation by salicylic acid [35], assuming that WHIRLY1 is already present in an inactive state. This pool of WHIRLY1 inactive in binding to *PR* gene promoters is presumably the pool that is located in the chloroplast. Another example for a chloroplast protein involved in pathogen defense is NRIP1. Infection of plants by tobacco mosaic virus was shown to induce its accumulation in the cytoplasm and nucleus [45]. The involvement of chloroplasts in pathogen response reactions has received more attention after it became apparent that many pathogens attempt to intercept signaling from chloroplasts by deploying effectors that target the chloroplasts in order to dampen the release of retrograde stress signals [50]. Secreted effector proteins of the pathogenic bacterium *Pseudomonas syringae*, for example, have *N*-terminal sequences (PTP) that are predicted to allow their import into the chloroplasts of infected cells [9]. It is noteworthy that one protein, Hop U1, targets several chloroplast-localized RNA-binding proteins and thus suppresses plant innate immunity [51]. It is possible that such proteins secreted by pathogens are plastid-targeted in order to interfere with the retrograde signaling from plastids either by preventing the production of defense related second messengers or by inhibiting the release of sequestered nuclear regulators, thus preventing the cascade of events that would otherwise lead to defense reactions.

In the current literature, the discussion on plastid-to-nucleus retrograde signaling focuses strongly on light-induced coordination of gene expression upon de-etiolation. So far, there is no evidence that any of the dually targeted proteins is involved in the extensive expression changes in the nucleus following this drastic change in the plant’s environment. Rather, the above-mentioned examples point to responses to plastid-perceived stresses such as pathogen infection.

## 5. Most Plastid/Nucleus Targeted Proteins are Involved in Gene Expression

Plastid gene expression is regulated at different levels involving transcription initiation, splicing, editing and processing of RNAs and also translation. Most of the plastid/nucleus targeted proteins are involved in one or the other step of this complex process. An intimate involvement of RNA-binding proteins in retrograde signaling has received support by the recent finding of an unprecedented link between RNA-editing and plastid-to-nucleus signaling [53]. These authors showed that treatment with different plastid retrograde signal influencing agents such as norflurazon or lincomycin reduced the RNA editing efficiency of various plastid transcripts. These defects were apparently not a secondary effect of the down-regulation of *PPR* genes involved in RNA editing [53]. Although the role of RNA editing in signaling pathways has not yet been specified, this result demonstrates the complexity of the retrograde signaling process and emphasizes the central role of plastid gene expression [54] and involvement of nucleic acid binding proteins in retrograde signaling. Several of the proteins binding to DNA and/or RNA in chloroplasts such as WHIRLY1 are associated with nucleoids [55]. It is possible that for formation of a retrograde signal not the actual level of dually targeted DNA/RNA binding proteins in chloroplasts is important, but rather the relative distribution between a fraction bound to nucleoids and a free pool detectable in the stroma [55]. This scenario would imply that a certain amount of the stored proteins is, in fact, free for release and does not necessarily have a function in plastids. However, this does not preclude that another fraction of the plastid isoforms plays a role in the plastids, most likely in the various processes associated with gene expression located in plastid nucleoids.

Maize transposon mutants impaired in WHIRLY1 were reported to have a bleached phenotype [56] that was later attributed to an increase in illegitimate plastome recombination, which is indicative of a decrease in plastid DNA stability in the absence of WHIRLY1 [57,58]. While several studies [59] indicated that WHIRLY1 preferentially binds to DNA, two studies on maize and barley came to the conclusion that the association of WHIRLY1 to nucleoids is due to an association with intron-containing mRNAs rather than with the plastid DNA [55]. Similarly to WHIRLY1, MFP1 and the cpRNPs cp29B and cp31A (see above), several other dually targeted proteins are associated with the plastid DNA or their RNAs [12]. Some examples for dually targeted proteins having a function associated with plastid DNA or RNA can be also found in the proteomes of nucleoids or so-called transcriptionally active chromosomes, e.g., PEND [31], HEMERA/pTAC12 [41,42] and SWIB-4 [48].

## 6. What is the Evidence for Protein Release from Plastids?

Several of the dually targeted proteins have the same molecular mass in plastids and the nucleus. The first hint that the nuclear isoforms of plastid/nucleus targeted proteins do not arise by twin targeting (*i.e.*, that a protein is either imported into one or the other compartment) or alternative splicing came from immunoblot analysis. The SEBF protein of potato [23], the WHIRLY1 protein [33] and a SET domain protein [38] possess nuclear and plastid isoforms of similar size. In all these cases the sizes correspond to those of the processed mature plastid isoforms lacking the plastid target peptide (PTP). Terasawa and Sato [31] were able to show that the accumulation of the nuclear isoform of the PEND protein [31] was dependent on the deletion of exactly those *N*-terminal 15 amino acids that are cleaved off during chloroplast import. The authors therefore suggested that the isoforms in both compartments are identical and that the mature form in chloroplasts might be translocated to the nucleus upon certain stimuli. Interestingly, the export of some proteins like yeast fumarase [60] and the human DEAD-box helicase MDDX28 [61] from mitochondria has also been shown to occur only after the cleavage of the *N*-terminal target sequence in the mitochondrial matrix. Following export, an import into the nuclear compartment has been demonstrated in case of mitochondrial MDDX28 [61].

Recently, an experimental approach provided unambiguous evidence for the existence of similar retrograde translocation pathways in plastids. For this, a recombinant tagged WHIRLY1 protein was expressed from a transgene that was integrated into the plastid genome [17]. This protein was detected in the nucleus by virtue of its HA tag using immunological methods. Moreover, a change in expression of two of WHIRLY1’s nuclear target genes was demonstrated, providing irrevocable evidence for the release of the recombinant protein from the plastids.

Given the fact that most cellular mechanisms of fundamental significance are conserved among eukaryotic cells and even between eukaryotes and prokaryotes and that specific peptide and protein export machineries exist in prokaryotes as well as in mitochondria of yeast and mammalian cells, it comes as no surprise that the existence of a similar export across the chloroplast envelope membrane has been proposed already some time ago [62]. The assumption of protein retrograde translocation recently gained momentum when the hypothesis was presented that proteins could be sequestered inside the chloroplasts in order to be released upon specific stimuli whereupon they can initiate nuclear responses [12,31].

## 7. Putative Release Pathways for Proteins Sequestered in Plastids

So far, the mechanism(s) by which proteins can be released from plastids remain elusive, but a number of possibilities have been brought up in the past, that will be summarized here.

### 7.1. Stromule Tip Shedding

Stromules are tubular extensions by individual plastids that are filled with stroma [63]. They display considerable dynamics in their extension and contraction [64–66] and appear to form physical and physiological bridges between different plastids [67] and between plastids and the nucleus [68]. This led to the suggestion that they might be facilitators for the exchange of molecules between the different compartments. Although an exchange of proteins via the stromules was recently strongly contested [69], the question has not been unambiguously solved. In any case, the recent study does not exclude that proteins other than the one under investigation might be translocated by stromules.

Stromule formation has been shown to be dependent of tissue and cell type [70], and the abundance and length of stromules seems to be inversely correlated with the size of plastids [71]. They were also shown to be induced by stress treatments acting through abscisic acid [72] making them excellent candidate structures for the transfer of stress induced plastid signals. A phenomenon that makes stromules intriguing candidates for plastid protein export is the shedding of protein-containing double membrane bound vesicles from their tips [65]. So far, such vesicles were described to be destined for degradation in the vacuolar compartment [73,74], but it cannot be excluded that such vesicles also fuse with compartments other than the vacuole, like the ER or Golgi, enabling a redistribution of the contents of the vesicles within the cell. So far lifetime imaging using cutting edge fluorescent markers has to our knowledge not provided hints for or against any of these possibilities.

### 7.2. Direct Membrane Contacts and Vesicle Budding from the Plastid Envelope Membrane

Physically tight membrane contact sites (MCS’s) between the plastid membranes and the ER membranes [75] that could facilitate or stabilize intercompartmental contacts have been proposed and are also under discussion as potential sites for the exchange of lipids and other metabolites. Whether such sites could also be used to shuttle proteins between compartments, specifically between the plastids and the nucleus, has not been tested.

**Figure 2:**
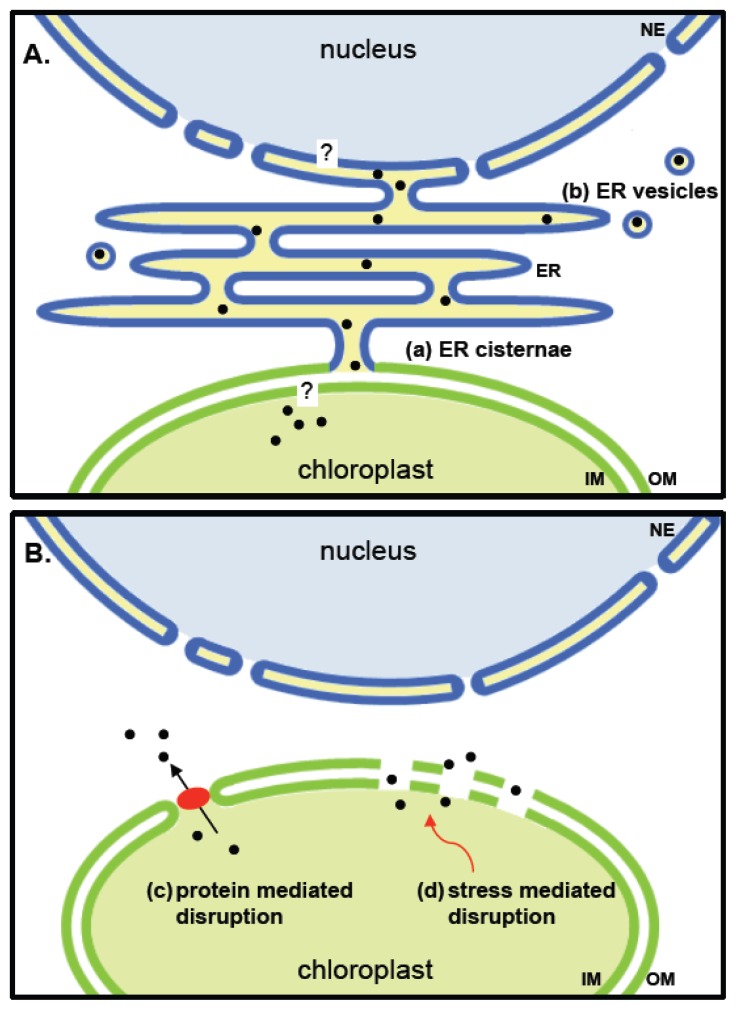
Selected mechanisms of protein translocation from chloroplasts to the nucleus. (**A**) Putative ER mediated transfer of plastid proteins to the nucleus. A periplasmic space formed by ER cisternae and intermembrane space of plastids has been observed under certain conditions (see text). Proteins from the stroma of plastids would need to transverse a single membrane to become included in this space, which is continuous with the envelope of nuclei. To enter the nucleus, proteins would need to cross the inner membrane of the nuclear envelope; (**B**) Hypothetical release of proteins directly into the cytoplasm. Transient pores might be formed by activity of proteins such as TGD2 being involved in lipid exchange between ER and plastids [90]. Small disruptions in the membrane leading to a leakiness of chloroplasts might occur upon stress. Black dots, released plastidic proteins; red ellipse, protein complex which mediates membrane permeability; NE, nuclear envelope; ER, endoplasmic reticulum; IM, inner membrane; OM, outer membrane.

At certain stages of chloroplast development, ER cisternae were found to form a sheath around plastids and the membranes even became continuous with the outer envelope membrane of plastids [76,77] (see Figure 2A). It is not unlikely that vesicles can be formed from such an ER-like periplastic space. Such vesicles would consist of only one surrounding membrane (Figure 2A) and would thereby differ from the vesicles formed by tip shedding of stromules. Vesicles surrounded by a single envelope membrane are a common form of communication for both bacteria and mitochondria [78,79]. In bacteria, vesicles can include proteins, toxins and DNA [80]. Protein export by vesicles was also observed in the symbiotically living cyanobacterium *Azolla microphylla* where such vesicles are released into the extracellular space [81]. Mitochondria derived vesicles were shown to contain specific cargo proteins indicating a selectivity of protein sorting into vesicles [82].

### 7.3. Channels or Retrograde Protein Transporters

In yeast mitochondria, peptide transport is mediated by a member of the ATP-binding cassette (ABC) transporter family, MDL1 [83,84], that is a homologue of the ER-located TAP protein. TAP is known to transport peptides into the ER lumen [85]. Unlike in mitochondria, no envelope-localized transport systems for peptide or protein export have been detected in plastids of higher plants so far, so their existence (see model in Figure 2B) is still hypothetical.

Protein secretion is a known phenomenon in bacteria that has actually been conserved in the chloroplasts. The general secretory (SEC) pathway and the twin-arginine translocation (TAT) pathway of bacteria [62,86] are both found in the thylakoid membranes of plastids where they are responsible for the import of proteins into the thylakoid lumen. This, by definition, is export from the chloroplast stroma, albeit to a different extraplastidial compartment. Interestingly, a dual localization of the Sec pathway in thylakoids as well as envelope membranes also of cyanelles (the plastids of glaucocystophytes) has been reported very recently [87]. Even if these pathways do not play a role in the envelope membrane of higher plant plastids, as it is believed at the moment, the detection of novel bacterial transport systems [88] and the improvement of whole plant genomic data increase the chance of finding other putative candidates through plant-prokaryote phylogenomics [89].

### 7.4. Changes in the Permeability of the Plastid Envelope

Most instances of retrograde translocation from the plastid and mitochondrial compartments—in plants likewise as in yeast or mammalian cells—are connected to stress or pathogen attack (Figure 1) and often result in hypersensitive responses and programmed cell death, processes known to be associated with membrane leakage [91]. Many proteins released under such conditions from mitochondria are located in the intermembrane space and, therefore, have to cross only one membrane. In contrast, the chloroplast proteins for which a translocation to the nucleus is under discussion are mostly located in the stroma and would have to transverse two membranes. The outer membrane of chloroplasts is, however, much more permeable than the inner membrane and might also get easily disrupted especially under situations of stress [92]. Even in the absence of stress, a protein-mediated disruption might be feasible (Figure 2B). The trigalactosyldiacylglycerol protein TGD2, which was found to be involved in the ER for chloroplast lipid transport, was shown to disrupt lipid bilayers. The protein is part of a larger complex in the chloroplast envelope and is anchored with its termini in both membranes [90]. It has not yet been investigated whether during lipid transfer transient pores big enough to let proteins pass are formed.

## 8. Outlook

Several proteins were described to be dually located in plastids and the nucleus. For one of them—WHIRLY1—the transfer from chloroplasts to the nucleus has been experimentally demonstrated employing transplastomic tobacco plants synthesizing the tagged protein inside the organelle. The transplastomic plants enable to study the export of the protein without interference with the import of proteins. Several scenarios for protein exports from plastids are possible. If the export is stimulated by stress related factors such as reactive oxygen species, the membrane might get leaky. ER-chloroplast contacts could be involved in the transfer of proteins from plastids to the nucleus. Tagged proteins synthesized in the plastid will allow investigating the pathway from plastids to the nucleus.

Although there is yet no information on development related changes in the dynamic abundances of dual targeted regulatory proteins in the different compartments, it is likely that plastids of different developmental stages have specific sets of regulatory proteins destined for the nucleus reflecting their functional situation within a given tissue and at a specific developmental stage. Proteome analyses with purified plastids and nuclei at different stages of plant development are required to address this question.

## Figures and Tables

**Table 1:** List of proteins that are targeted to the plastids (p) and the nucleus (n).

First described	Protein name(s)	Protein function	References	Release from plastids
1995	cp29B (p) SEBF (n)	RNA-binding protein transcriptional repressor	[21,27] [23]	not investigated
1995	cp31A (p) STEP1 (n)	RNA-binding protein telomere-binding	[21,27] [24,28]	not investigated
1996	MFP1 (n) MFP1 (p)	matrix attachment region binding nucleoid associated protein	[22] [26]	not investigated
1996	GSBF1 (n) PEND (p)	transcription factor nucleoid associated protein	[29] [30]	possible [31]
1997	DHFR (p + n)	dihydrofolate reductase/thymidylate synthase	[19]	no [19]
2004	LEM1 (p + n)	unknown (homologous to plastid ribosomal protein PRPS9)	[32]	not investigated
2005	WHIRLY1 (p) WHIRLY1 (n)	DNA + RNA binding; DNA maintenance transcriptional activator; telomere-binding	[33,34] [35,36]	yes [17]
2005	CDT1 (p) CDT1 (n)	plastid division (interaction with Arc6) DNA replication	[37] [37]	not investigated
2006	ATXR5 (p + n)	control of cell cycle and DNA replication in the nucleus; plastid function unknown	[38]	not investigated
2006	NtWIN4 (p) NtWIN4 (n)	induction of hypersensitive cell death transcriptional repressor	[39] [39]	no [40]
2006	pTAC12 (p) HEMERA (n)	nucleoid associated protein phytochrome signalling	[41] [42]	not investigated
2007	At2g44940 (p + n)	transcription factor with AP2 DNA binding motif	[43]	not investigated
2008	IPT3 (p + n)	cytokinin biosynthesis in plastids; nuclear function unknown	[44]	no [44]
2008	NRIP1 (p + n)	rhodanese sulfur transferase; immune receptor recognition; plastid function unknown	[45]	possible [45]
2011	SIB1, SIB2 (p + n)	proteins binding to Sigma factor1 of plastid encoded RNA-polymerase	[46]	not investigated
2012	ANAC102 (p + n)	NAC transcription factor	[47]	not investigated
